# Disinfectants in Homœopathic Practice

**Published:** 1887-09

**Authors:** 


### HOMŒOPATHIC PHYSICIAN,

A MONTHLY JOURNAL OF MEDICAL SCIENCE.

"If our school ever gives up the strict inductive method of Hahnemann, we are lost, and deserve only to be mentioned as a caricature in the history of medicine."—CONSTANTINE HERING.

Vol. VII. SEPTEMBER, 1887. No. 9.

### EDITORIAL NOTES.

DISINFECTANTS IN HOMŒOPATHIC PRACTICE.—In its broadest sense the term disinfectant is used to designate measures or chemicals used to deodorize bad smells and to *prevent* spread of disease. In their antiseptic features disinfectants are valuable; as deodorizers they are useless and interfere with homœopathic prescribing. Used to prevent the spread of cholera, typhoid fever, etc.; used to promote cleanliness and thereby to remove the causes of disease, disinfectants are invaluable, though it must be frankly stated that as antiseptics disinfectants are often found unreliable.

As deodorizers these measures have little or no place in homœopathic practice. This may seem a very bold and startling statement, but it is true. The color, the odor and appearance, etc., of a patient's discharges are to be taken into consideration in selecting the remedy. They are as much symptoms of the patient as are his aches and pains; if these be masked or changed by use of deodorizers *before* the physician has noted these peculiarities, then he has lost an important guide for the choice of his remedy. We do not mean to assert that these discharges, etc., must be kept in the sick-room, or in any place where they can do harm; but we do mean to say that the patient's discharges are important in their color, odors, etc., and these should not be destroyed until the attending physician has examined them. It is also a fashion among some to cleanse ulcers, etc., with "mild disinfectants," which, of course, must change their appearance and do harm.

In the pathogeneses of our drugs we have given the odor of the stools, of the perspiration, etc. These are useful symptoms, and, moreover, these medicines will not only cure the patient, but they will remove the offensive odors, and do it promptly. Many of our remedies are first-class deodorizers.

